# Menstrual Cycle in Trauma-Related Disorders: A Mini-Review

**DOI:** 10.3389/fgwh.2022.910220

**Published:** 2022-05-30

**Authors:** Eveline Mu, Elizabeth H. X. Thomas, Jayashri Kulkarni

**Affiliations:** Monash Alfred Psychiatry Research Centre, Central Clinical School, Monash University and The Alfred Hospital, Melbourne, VIC, Australia

**Keywords:** PTSD, BPD, CPTSD, trauma, estradiol, progesterone

## Abstract

Fluctuations of sex hormones across the menstrual cycle have been linked to exacerbation of symptoms of psychiatric disorders. Women diagnosed with trauma-related disorders such as post-traumatic stress disorder (PTSD) and borderline personality disorder (BPD) have reported worsening of mood symptoms at various phases of their menstrual cycle. There is also considerable overlap between PTSD, BPD, and complex-PTSD (cPTSD) symptoms, suggesting similar biological underpinnings. This mini-review examines the influence of sex hormones and the menstrual cycle on PTSD, BPD, and cPTSD symptoms, and discusses the involvement of the hypothalamic-pituitary-adrenal (HPA) axis. We review literature showing that PTSD and BPD symptoms fluctuate with the menstrual cycle, though the effect of the menstrual cycle phase appears to be inconsistent, warranting future research. Based on the reported phasic vulnerability in individuals with PTSD and BPD, it is plausible to assume that women diagnosed with the newly introduced cPTSD may experience similar difficulties. However, no study to date has addressed this. This review highlights the importance of considering an individual's trauma history as it may influence symptom severity and diagnosis, and the phase of the menstrual cycle at the time of diagnosis. This review also highlights that additional work is needed to clarify the influence of estradiol and progesterone fluctuations on trauma-related symptoms, especially in cPTSD. Continued research on menstrual cycle vulnerability and trauma will lead to better informed management and treatment of PTSD, BPD, and cPTSD.

It is well-documented that the cyclical rise and fall of sex hormones across the menstrual cycle impacts symptoms of psychiatric disorders. The menstrual cycle typically lasts 23–35 days with four stages (1); menstruation, the follicular phase, ovulation, and the luteal phase. The menstrual cycle is characterized by fluctuations in gonadal hormones estrogen (in the form of estradiol) and progesterone in the central nervous system (CNS) that are driven by changes in the brain gonadotropin levels. Menstruation signifies the start of the menstrual cycle, and is when estradiol and progesterone are at their lowest. Menstruation lasts ~5 days, and is followed by the follicular phase. During this phase, estradiol increases to trigger the release of follicle stimulating hormone (FSH), which stimulates follicle (egg) development. The increase in estradiol also stimulates growth of the endometrium (uterus lining). Estradiol continues to rise to a peak, triggering increased production of luteinizing hormone (LH). The rapid surge in LH causes the release of the egg, known as ovulation. At this point, there is a rapid drop in estradiol. Finally, there is the luteal phase, where there is an increase in progesterone and estradiol to support early pregnancy. If there is no egg fertilization, progesterone and estradiol decrease; this decline causes premenstrual symptoms and triggers the onset of menses.

Healthy women have reported increased mental ill health symptoms related to depression and anxiety during the late luteal and early follicular phases (2). Menstrual cycle-related mood fluctuations have also been observed across a number of mental health disorders (3). Emerging studies suggest that menstrual cycle effects are evident in individuals with trauma-related disorders such as post-traumatic stress disorder (PTSD) (4, 5) and borderline personality disorder (BPD) (6, 7). In particular, childhood traumas including physical, sexual, and emotional abuse/and or neglect are very common in PTSD and BPD (8–10). It is estimated that 15 to 82.5% of individuals with PTSD have experienced childhood trauma (11–13), and 58 to 91% for individuals with BPD (14–16).

Both PTSD and BPD are more common in women (17–19), suggesting that ovarian steroid hormones estradiol and progesterone influence the expression of PTSD/BPD symptoms (20). In fact, the hormonal changes that occur across the menstrual cycle could biologically explain the fluctuation of symptoms observed in women with PTSD and BPD (5, 6, 21). This idea is strengthened with the known overlap in symptomology (particularly anger and irritability) between PTSD (22, 23) and BPD (24, 25), and premenstrual dysphoric disorder (PMDD). PMDD is a diagnostic classification for women who have significant functional impairment caused by moderate to severe premenstrual symptoms (17). The overlap is said to be greater between the trauma-related disorders and PMDD than PMDD and major depressive disorder (24, 26). PMDD has also been shown to overlap in symptomology with complex PTSD (cPTSD), another mental health disorder strongly associated with trauma exposure (27, 28). Furthermore, research indicates an overlap between PTSD, BPD and cPTSD (29).

This mini-review will now explore the influence of sex hormones and the menstrual cycle on PTSD, BPD, and cPTSD symptoms, and discuss potential underlying mechanisms.

## Post-Traumatic Stress Disorder (PTSD)

PTSD, recently re-classified as a trauma- and stressor-related disorder, is a psychiatric disorder that develops after exposure to a traumatic event. The Diagnostic and Statistical Manual of Mental Disorders (DSM-5) criteria list four PTSD symptom sub-categories: intrusion, avoidance, negative alterations in cognitions and mood, and marked alterations in arousal and reactivity. Similarly, the International Classification of Diseases (ICD-11) lists three PTSD symptom clusters: re-experiencing of the trauma, avoidance of trauma reminders, and heightened sense of threat.

In the general population, there are higher rates of PTSD in women (10–12%) than in men (5–6%), with women being two to three times more likely to develop PTSD (30). In addition, women have also reported greater PTSD symptom severity (31) than men. The gender differences in both prevalence and symptom severity suggest underlying sex-specific mechanisms influencing both the etiology and perpetuation of PTSD.

## PTSD Symptoms Across the Menstrual Cycle

Estradiol and progesterone levels at the time of trauma exposure have been suggested to contribute to the development of PTSD symptoms. For example, women with PTSD who experience trauma during the mid-luteal phase of their cycle have a greater number of flashbacks (32). Another study observed that women who refused emergency contraception following sexual assault had increased post-traumatic stress symptoms compared to those that took emergency contraception, or were already using hormonal contraceptives (33). While there are many severe psychological reactions following sexual assault, it is important to note that both emergency and standard contraception suppress progesterone and estradiol synthesis, and together with the findings of Bryant et al. (32), these suggest that low endogenous progesterone and/or estradiol has a protective effect in the etiology of PTSD, particularly in terms of preventing memory over-consolidation.

Not only do sex hormones during time of trauma exposure play a role in symptom presentation, but a recent review by Nillni et al. (5) observed that PTSD symptoms fluctuate alongside the menstrual cycle. For example, exacerbation of intrusive flashbacks appear to occur during the mid-luteal phase (when estradiol and progesterone are high), with women 4.89 times more likely to have flashbacks during this phase compared to other menstrual cycle phases (32).

Conversely, trauma-exposed women have higher PTSD symptoms during low-estradiol phases of their cycle (34). In a sample of trauma-exposed women with and without PTSD, an increase in overall psychological symptoms, as well as depression and phobic anxiety specifically, was observed in the early follicular menstrual cycle phase compared to mid-luteal phase (35). When the groups were explored separately, phobic anxiety again increased in the early follicular phase in women with PTSD, but not in women without PTSD.

An effective behavioral treatment for PTSD is exposure therapy, which involves controlled exposure to a situation or stimulus with the purpose of reducing anxiety and distress over time. Fear extinction (i.e., the decrease and cessation of a fear response through exposure therapy) appears to be influenced by estradiol levels, though findings in women with PTSD are inconsistent when exploring this by menstrual cycle phase. Greater extinction retention in females in high compared to low estradiol groups have been observed in animal studies (36) and in healthy women (37). Research suggests that estradiol levels modulate extinction memory in women with PTSD (38), though there have been inconsistencies when exploring the nature of this relationship. While Pineles et al. (39) observed poorer extinction retention during the mid-luteal phase (when there would be an increased in estradiol), other studies have reported heightened startle responses during fear conditioning in PTSD-diagnosed women with low estradiol (40). However, Glover et al. (40) did not account for menstrual cycle phase at the time of blood sampling, which may explain the difference in findings.

Current literature indicates a phasic vulnerability in women with PTSD, with different symptoms exacerbated at different points of the menstrual cycle. It remains unclear as to whether high levels of estradiol and progesterone are protective or increase vulnerability in the maintenance of PTSD symptoms in women. It should be noted that some studies have observed stability in symptoms across the menstrual cycle, and no associations between estradiol and progesterone levels and anxiety sensitivity (41). Again, this highlights that estradiol and progesterone affects distinct trauma-related symptoms differently, warranting further research.

## Borderline Personality Disorder (BPD)

Borderline personality disorder (BPD) is a chronic mental condition characterized by rapidly shifting emotional and behavioral symptoms, and difficulties maintaining stable interpersonal relationships. Common BPD features include low self-esteem, fear of abandonment, identity disturbances, impulsive behaviors, and maladaptive coping strategies (17). While BPD is not currently considered an exclusively post-traumatic phenomenon in the DSM-5, majority of individuals with BPD have experienced childhood physical (42, 43), sexual (42–44) or emotional (16, 45) abuse. The prevalence rates of early life trauma in BPD range from 58 to 91% (14, 16, 46), and 75%of people diagnosed with BPD are women (17).

## BPD Symptoms Across the Menstrual Cycle

BPD is characterized by impulsivity, and cyclical changes in ovarian hormones have been associated with several impulsive-related behaviors. Earlier studies have indicated a premenstrual increase in suicidal behavior (47, 48) and severity of suicide intent (49, 50), binge eating in patients with bulimia (51–53), and worsening of depressive symptoms (7, 24) in patients with BPD.

While women with BPD often report that affective and impulsive symptoms worsen premenstrually, empirical evidence has been inconsistent. An early small prospective study of women with BPD found no association between the phase of the menstrual cycle and affective and impulsivity (7). However, emerging work suggests potential menstrual cycle effects on BPD symptom exacerbation. Specifically, two studies revealed a link between cyclical menstrual changes and BPD-like features in non-clinical samples (6, 21). Two studies demonstrated menstrual cycle-related changes in BPD symptoms in unmedicated women with regular menstrual cycles in clinical samples (24, 54). Considering these four studies, it appears that individuals with BPD had a clear pattern of perimenstrual (i.e., late luteal phase to day 3 of menses) exacerbation, with symptoms of irritability and anger appearing in the luteal phase and peaking in the perimenstrual phase. Symptoms of depression and hopelessness were heightened in the perimenstrual phase and persisted into the follicular phase. Symptoms improved and were least apparent in the ovulatory phase—where women had higher levels of systemic estradiol.

Aggression can be classified into reactive and proactive forms, with both types being exacerbated in different menstrual phases. A recent study examined the differential effects of menstrual cycle changes on reactive and proactive aggression in individuals with BPD (54). Reactive aggression was defined as an uncontrolled outburst of anger, while proactive aggression was defined as planned aggression or manipulation (55). The authors revealed that anger, anger expressions, and aggression were typically lowest in the ovulatory phase, although proactive aggression was increased during the ovulatory phase. In the mid-luteal phase (defined as 18–21 days after the start of menstruation), anger and irritability were elevated and reactive aggression peaked. In the perimenstrual phase, anger and irritability peaked, anger against self-increased, and reactive aggression began to decrease. Eventually, anger returned to a “baseline” level in the follicular phase. Findings from this study imply that it is also important to consider the type of aggression in reproductive aged women with BPD as reactive and proactive aggressions increase during the different phases of their menstrual cycles.

Altogether, the current literature suggests that sensitivity to gonadal hormone fluctuations in the CNS could influence the etiology and maintenance of BPD, and individuals with BPD may experience symptom changes in the type and severity of symptoms at different menstrual cycle phases. Generally, fewer BPD symptoms are experienced in the ovulatory period when CNS estradiol levels are high and BPD symptoms increase and peak around the mid-luteal and perimenstrual phases when CNS estradiol and progesterone levels are lower. There is a clear need for further research into understanding the relationship between ovarian hormones fluctuations across the menstrual cycle and BPD symptoms. Such research may well lead to more effective management of BPD with new gonadal hormone treatment strategies.

## Complex Post-Traumatic Disorder (CPTSD)

As described in the ICD-11, cPTSD consists of six symptoms clusters: the three core PTSD symptoms (re-experiencing of the trauma, avoidance of trauma reminders and heightened sense of threat) and one symptom from each of three disturbances of self-organization (DSO) symptom clusters (emotional dysregulation, interpersonal difficulties, and negative self-concept).

Similar to PTSD and BPD, the prevalence of cPTSD is higher in women, with all three symptom clusters also reported significantly more often (18). Despite this gender difference, there are no studies to our knowledge that have explored the influence of sex hormones and/or the menstrual cycle on cPTSD symptoms. Considering the substantial overlap in symptoms between PTSD, BPD, and cPTSD, the relationship between symptom exacerbations and fluctuations across the menstrual cycle in PTSD and BPD may extend to cPTSD. This is a significant gap in the literature that needs to be addressed.

## Discussion

### HPA Axis

While there are some differences between PTSD, BPD and cPTSD, the substantial symptom overlap indicates a similar underlying mechanism for these conditions (29). As trauma plays a key role in these disorders, it has been suggested that stress and subsequent alterations in the hypothalamic-pituitary-adrenal (HPA) axis may contribute to the aetiopathogenesis of these disorders (56–59).

The HPA axis links the CNS with peripheral tissues through the involvement of the hypothalamus, the pituitary, and the adrenals. This system of neuroendocrine pathways and feedback loops regulate a broad range of physiological processes, in particular mediating the stress response, and hence is consistently implicated in trauma-related conditions.

There is overlap in the neurochemistry and neurostructural changes observed in PTSD, BPD and cPTSD, with no clear differentiation between the three conditions in regards to HPA axis function or subsequent alterations (29). For instance, HPA axis overactivity has been implicated in BPD (60) and PTSD (56), and this dysfunction in both disorders is believed to closely mirror each other (58).

There is a complex bi-directional relationship between the HPA and hypothalamic-pituitary-gonadal (HPG) axes; the latter playing a key role in regulating reproduction by controlling gonadal hormones. The activation of one axis impacts the function of the other and vice versa (61). The HPA and HPG axes need to remain in equilibrium and factors that upset homeostasis, such as stress after trauma exposure, result in neuroendocrine disruption. This ultimately affects both the actions of the gonadal hormones such as estradiol and progesterone, as well as the endocrine hormones such as cortisol. This then contributes to menstrual cycle disruptions (62), which increases the risk of PTSD, BPD, and cPTSD.

### Limitations and Future Directions

Many studies, such as those dealing with BPD, lacked a control group comparison, making it difficult to determine whether the patterns observed were specific to BPD or if they were attributed simply to fluctuations in the menstrual cycle, regardless of psychological functioning (21, 24, 54). PTSD studies focused on broad classification of trauma-exposed women with and without PTSD, while most BPD studies did not consider trauma and those that did grouped women by trauma-exposed and not exposed. While group comparisons are important, studies should explore within-person symptom change, especially considering that menstrual cycles differ vastly in populations of women.

Future studies could explore the relationship between the type, severity and frequency of trauma and the exacerbation of trauma-related symptoms across the menstrual cycle. The timing of trauma is also an important factor that should be explored, since childhood trauma experienced as a toddler or pre-schooler, may have quite different impacts compared to trauma occurring at school-age or in adolescence. In addition, as there are no studies exploring cPTSD and the menstrual cycle, future studies in this area can model existing study designs in PTSD and BPD given the overlap in biological underpinnings and symptomatology.

## Implications AND Conclusion

This review provides a few practical and theoretical implications about the possible relationship between gonadal hormone fluctuations of the menstrual cycle and behavioral, emotional, and cognitive symptoms that are seen in conditions such as PTSD, BPD and cPTSD. These three psychiatric conditions are all caused, at least in part, by trauma. Hence, it is important to consider each individual's trauma history in general. We need to consider a broader definition of trauma that considers the stressful impact of poor bonding with a primary carer, emotional neglect, or invalidation, as well as actual instances of physical abuse and sexual trauma. It appears that an experience of trauma can significantly influence an individual's neurobiology. In reproductive aged women, studying the impact of both gonadal hormone fluctuations and trauma experiences on resultant psychiatric symptoms may provide new neurobiological insights. We highlight that menstrual cycle phases could influence an individuals' diagnosis, so clinicians' need to be mindful of the phase of menstrual cycle at the time of diagnosis or perform assessments at different time points to gain a better understanding of the psychiatric symptoms' presence and severity. Similarly, clinicians should consider the phase of the menstrual cycle at the time of trauma exposure as this can contribute to symptom severity.

In conclusion, the present review provides evidence that in women with trauma-based disorders, the fluctuations of gonadal hormones across the menstrual cycle appear to cause changes in psychiatric symptoms at different monthly time-points. Psychiatric symptoms of PTSD and BPD appear to vary markedly across the menstrual cycle in women who have experienced early life trauma. This suggests that trauma alters the brain's sensitivity to gonadal hormone fluctuations, resulting in marked worsening of psychiatric symptoms in the low estradiol menstrual cycle phases, and some improvement in higher estradiol phases. While the literature is lacking in cPTSD, one may assume that the alteration in brain sensitivity may extend to the cPTSD population. This review also highlights that additional work is needed to clarify the influence of estradiol and progesterone fluctuations on trauma-related symptoms. Continued research on menstrual cycle vulnerability and trauma will lead to better informed management and treatment of PTSD, BPD, and cPTSD.

## Author Contributions

EM and ET were equally involved in the conceptualization of the mini-review and draft of manuscript. All authors contributed to manuscript editing.

## Conflict of Interest

The authors declare that the research was conducted in the absence of any commercial or financial relationships that could be construed as a potential conflict of interest.

## Publisher's Note

All claims expressed in this article are solely those of the authors and do not necessarily represent those of their affiliated organizations, or those of the publisher, the editors and the reviewers. Any product that may be evaluated in this article, or claim that may be made by its manufacturer, is not guaranteed or endorsed by the publisher.

## References

[1] Reed BG Carr BR. The Normal Menstrual Cycle and the Control of Ovulation. In: Endotext. South Dartmouth, MA: MDText.com, Inc. (2000). 25905282

[2] Gonda X Telek T Juhász G Lazary J Vargha A Bagdy G. Patterns of mood changes throughout the reproductive cycle in healthy women without premenstrual dysphoric disorders. Prog Neuro Psychopharmacol Biol Psychiatry. (2008) 32:1782–8. 10.1016/j.pnpbp.2008.07.016 18721843

[3] Hendrick V Altshuler LL Burt VK. Course of psychiatric disorders across the menstrual cycle. Harv Rev Psychiatry. (1996) 4:200–7. 10.3109/10673229609030544 9384994

[4] Green SA Graham BM. Symptom fluctuation over the menstrual cycle in anxiety disorders, PTSD, and OCD: a systematic review. Arch Women's Mental Health. (2021) 25:71–85. 10.1007/s00737-022-01237-5 34668073

[5] Nillni YI Rasmusson AM Paul EL Pineles SL. The impact of the menstrual cycle and underlying hormones in anxiety and PTSD: what do we know and where do we go from here? Curr Psychiatry Rep. (2021) 23:1–9. 10.1007/s11920-020-01221-9 33404887 PMC8819663

[6] DeSoto MC Geary DC Hoard MK Sheldon MS Cooper L. Estrogen fluctuations, oral contraceptives and borderline personality. Psychoneuroendocrinology. (2003) 28:751–66. 10.1016/S0306-4530(02)00068-9 12812862

[7] Ziv B Russ MJ Moline M Hurt S Zendell S. Menstrual cycle influences on mood and behaviour in women with borderline personality disorder. J Pers Disord. (1995) 9:68–75. 10.1521/pedi.1995.9.1.68

[8] Brand B. Trauma and women. Psychiatr Clin N Am. (2003) 26:759–79. 10.1016/S0193-953X(03)00034-0 14563108

[9] Cattane N Rossi R Lanfredi M Cattaneo A. Borderline personality disorder and childhood trauma: exploring the affected biological systems and mechanisms. BMC Psychiatry. (2017) 17:221. 10.1186/s12888-017-1383-2 28619017 PMC5472954

[10] Golier JA Yehuda R Bierer LM Mitropoulou V New AS Schmeidler J. The relationship of borderline personality disorder to posttraumatic stress disorder and traumatic events. Am J Psychiatry. (2003) 160:2018–24. 10.1176/appi.ajp.160.11.2018 14594750

[11] Breslau N Wilcox HC Storr CL Lucia VC Anthony JC. Trauma exposure and posttraumatic stress disorder: a study of youths in urban America. J Urban Health. (2004) 81:530–44. 10.1093/jurban/jth138 15466836 PMC3455932

[12] Copeland WE Keeler G Angold A Costello J. Traumatic events and posttraumatic stress in childhood. Arch Gen Psychiatry. (2007) 64:577–84. 10.1001/archpsyc.64.5.577 17485609

[13] Giaconia RM Reinherz Z H Silverman B A Pakiz B. Trauma and posttraumatic stress disorder in a community population of older adolescents. J Am Acad Child Adolesc Psychiatry. (1995) 34:1369–80. 10.1097/00004583-199510000-00023 7592275

[14] Soloff PH Lynch KG Kelly TM. Childhood abuse as a risk factor for suicidal behaviour in borderline personality disorder. J Pers Disord. (2002) 16:201–14. 10.1521/pedi.16.3.201.22542 12136678

[15] Zanarini MC. Childhood experiences associated with the development of borderline personality disorder. Psychiatr Clin North Am. (2000) 23:89–101. 10.1016/S0193-953X(05)70145-3 10729933

[16] Zanarini MC Gunderson JG Marino MF Schwartz EO Frankenburg FR. Childhood experiences of borderline patients. Compr Psychiatry. (1989) 30:18–25. 10.1016/0010-440X(89)90114-4 2924564

[17] American Psychiatric Association. Diagnostic and Statistical Manual of Mental Disorders: DSM-5. Washington, DC: American Psychiatric Association (2013).

[18] Knefel M Lueger-Schuster B. An evaluation of ICD-11 PTSD and complex PTSD criteria in a sample of adult survivors of childhood institutional abuse. Eur J Psychotraumatol. (2013) 4:22608. 10.3402/ejpt.v4i0.22608 24312721 PMC3851534

[19] Olff M. Sex and gender differences in post-traumatic stress disorder: an update. Eur J Psychotraumatol. (2017) 8:1351204. 10.1080/20008198.2017.135120415920002

[20] Grant BF Chou SP Goldstein RB Huang B Stinson FS Saha TD. Prevalence, correlates, disability, and comorbidity of DSM-IV BPD: results from the Wave 2 National Epidemiologic Survey on Alcohol and Related Conditions. J Clin Psychiatry. (2008) 69:533–45. 10.4088/jcp.v69n0404 18426259 PMC2676679

[21] Eisenlohr-Moul TA DeWall CN Girdler SS Segerstrom SC. Ovarian hormones and borderline personality disorder features: preliminary evidence for interactive effects of estradiol and progesterone. Biol Psychol. (2015) 109:37–52. 10.1016/j.biopsycho.2015.03.016 25837710 PMC4516641

[22] Pilver CE Levy BR Libby DJ Desai RA. Posttraumatic stress disorder and trauma characteristics are correlates of premenstrual dysphoric disorder. Arch Women's Mental Health. (2011) 14:383–93. 10.1007/s00737-011-0232-4 21786081 PMC3404806

[23] Wittchen HU Perkonigg A Pfister H. Trauma and PTSD–an overlooked pathogenic pathway for premenstrual dysphoric disorder? Arch Women's Mental Health. (2003) 6:293–7. 10.1007/s00737-003-0028-2 14628182

[24] Eisenlohr-Moul TA Schmalenberger KM Owens SA Peters JR Dawson DN Girdler SS. Perimenstrual exacerbation of symptoms in borderline personality disorder: evidence from multilevel models and the Carolina Premenstrual Assessment Scoring System. Psychol Med. (2018) 48:2085–95. 10.1017/S0033291718001253 29804553 PMC6436806

[25] Schiller CE Johnson SL Abate AC Schmidt PJ Rubinow DR. Reproductive steroid regulation of mood and behavior. Compr Physiol. (2016) 6:1135–60. 10.1002/cphy.c150014 27347888 PMC6309888

[26] Freeman EW Halberstadt SM Rickels K Legler JM Lin H Sammel MD. Core symptoms that discriminate premenstrual syndrome. J Women's Health. (2011) 20:29–35. 10.1089/jwh.2010.2161 21128818 PMC3017419

[27] Grewal JK Kulkarni J. Complex post-traumatic stress disorder and pre-menstrual dysphoric disorder. Aust N Z J Psychiatry. (2022) 56:203–4. 10.1177/00048674211025627 34134539

[28] Robertson E Thew C Thomas N Karimi L Kulkarni J. Pilot data on the feasibility and clinical outcomes of a nomegestrol acetate oral contraceptive pill in women with premenstrual dysphoric disorder. Front Endocrinol. (2021) 12:704488. 10.3389/fendo.2021.704488 34630323 PMC8498579

[29] Kulkarni J. Complex PTSD–a better description for borderline personality disorder? Australasian Psychiatry. (2017) 25:333–35. 10.1177/1039856217700284 28347146

[30] Olff M Langeland W Draijer N Gersons BP. Gender differences in posttraumatic stress disorder. Psychol Bull. (2007) 133:183. 10.1037/0033-2909.133.2.183 17338596

[31] Ullman SE Filipas HH. Gender differences in social reactions to abuse disclosures, post-abuse coping, and PTSD of child sexual abuse survivors. Child Abuse Neglect. (2005) 29:767–82. 10.1016/j.chiabu.2005.01.005 16051351

[32] Bryant RA Felmingham KL Silove D Creamer M O'Donnell M McFarlane AC. The association between menstrual cycle and traumatic memories. J Affect Disord. (2011) 131:398–401. 10.1016/j.jad.2010.10.049 21093927

[33] Ferree NK Wheeler M Cahill L. The influence of emergency contraception on post-traumatic stress symptoms following sexual assault. J Forensic Nurs. (2012) 8:122–30. 10.1111/j.1939-3938.2012.01134.x 22925127 PMC3430967

[34] Rieder JK Kleshchova O Weierich MR. Estradiol, stress reactivity, and daily affective experiences in trauma-exposed women. Psychol Trauma Theory Res Pract Policy. (2021) 10.1037/tra0001113. [Epub ahead of print]. 34726450 PMC9046469

[35] Nillni YI Pineles SL Patton SC Rouse MH Sawyer AT Rasmusson AM. Menstrual cycle effects on psychological symptoms in women with PTSD. J Trauma Stress. (2015) 28:1–7. 10.1002/jts.21984 25613589

[36] Milad MR Igoe SA Lebron-Milad K Novales J. Estrous cycle phase and gonadal hormones influence conditioned fear extinction. Neuroscience. (2009) 164:887–95. 10.1016/j.neuroscience.2009.09.011 19761818 PMC2783784

[37] Milad MR Zeidan MA Contero A Pitman RK Klibanski A Rauch SL. The influence of gonadal hormones on conditioned fear extinction in healthy humans. Neuroscience. (2010) 168:652–8. 10.1016/j.neuroscience.2010.04.030 20412837 PMC2881679

[38] Hammoud MZ Foa EB Milad MR. Oestradiol, threat conditioning and extinction, post-traumatic stress disorder, and prolonged exposure therapy: a common link. J Neuroendocrinol. (2020) 32:e12800. 10.1111/jne.12800 31595559

[39] Pineles SL Nillni YI King MW Patton SC Bauer MR Mostoufi SM. Extinction retention and the menstrual cycle: different associations for women with posttraumatic stress disorder. J Abnorm Psychol. (2016) 125:349. 10.1037/abn0000138 26866677

[40] Glover EM Jovanovic T Mercer KB Kerley K Bradley B Ressler KJ. Estrogen levels are associated with extinction deficits in women with posttraumatic stress disorder. Biol Psychiatry. (2012) 72:19–24. 10.1016/j.biopsych.2012.02.031 22502987 PMC3675159

[41] Nillni YI Arditte Hall KA Langdon KJ Pineles SL. Examination of the stability of the anxiety sensitivity index across the menstrual cycle in trauma-exposed women with and without PTSD. Anxiety Stress Coping. (2020) 33:115–21. 10.1080/10615806.2019.1660322 31455152 PMC6910958

[42] Ogata SN Silk KR Goodrich S Lohr NE Westen D Hill EM. Childhood sexual and physical abuse in adult patients with borderline personality disorder. Am J Psychiatry. (1990) 147:1008–12. 10.1176/ajp.147.8.1008 2375434

[43] Westen D Ludolph P Misle B Ruffins S Block J. Physical and sexual abuse in adolescent girls with borderline personality disorder. Am J Orthopsychiatry. (1990) 60:55–66. 10.1037/h0079175 2305845

[44] McLean LM Gallop R. Implications of childhood sexual abuse for adult borderline personality disorder and complex posttraumatic stress disorder. Am J Psychiatry. (2003) 160:369–71. 10.1176/appi.ajp.160.2.369 12562587

[45] Westbrook J Berenbaum H. Emotional awareness moderates the relationship between childhood abuse and borderline personality disorder symptom factors. J Clin Psychol. (2017) 73:910–21. 10.1002/jclp.22389 27701740

[46] Martín-Blanco A Soler J Villalta L Feliu-Soler A Elices M Pérez V. Exploring the interaction between childhood maltreatment and temperamental traits on the severity of borderline personality disorder. Compr Psychiatry. (2014) 55:311–18. 10.1016/j.comppsych.2013.08.026 24262124

[47] Owens SA Eisenlohr-Moul T. Suicide risk and the menstrual cycle: a review of candidate RDoC mechanisms. Curr Psychiatry Rep. (2018) 20:106. 10.1007/s11920-018-0962-3 30293097 PMC6844188

[48] Sein Anand J Chodorowski Z Ciechanowicz R Wiśniewski M Pankiewicz P. The relationship between suicidal attempts and menstrual cycle in women. Przegl Lek. (2005) 62:431–3. 16225087

[49] Diamond SB Rubinstein AA Dunner DL Fieve RR. Menstrual problems in women with primary affective illness. Compr Psychiatry. (1976) 17:541–8. 10.1016/0010-440X(76)90036-5 986926

[50] Smith A Miller S Bodell L Ribeiro J Joiner T Maner J. Cycles of risk: associations between menstrual cycle and suicidal ideation among women. Pers Individ Dif. (2015) 74:35–40. 10.1016/j.paid.2014.09.043

[51] Gladis MM Walsh TB. Premenstrual exacerbation of binge eating in bulimia. Am J Psychiatry. (1987) 144:1592–5. 10.1176/ajp.144.12.1592 3688285

[52] Klump KL Keel PK Culbert KM Edler C. Ovarian hormones and binge eating: exploring associations in community samples. Psychol Med. (2008) 38:1749–57. 10.1017/S0033291708002997 18307829 PMC2885896

[53] Schoofs N Chen F Bräunig P Stamm T Krüger S. Binge eating disorder and menstrual cycle in unmedicated women with bipolar disorder. J Affect Disord. (2011) 129:75–8. 10.1016/j.jad.2010.08.016 20869775

[54] Peters JR Owens SA Schmalenberger KM Eisenlohr-Moul TA. Differential effects of the menstrual cycle on reactive and proactive aggression in borderline personality disorder. Aggress Behav. (2020) 46:151–61. 10.1002/ab.21877 31957896 PMC7458153

[55] Poulin F Boivin M. Reactive and proactive aggression: evidence of a two-factor model. Psychol Assess. (2000) 12:115–22. 10.1037/1040-3590.12.2.115 10887757

[56] Dunlop BW Wong A. The hypothalamic-pituitary-adrenal axis in PTSD: pathophysiology and treatment interventions. Prog Neuro Psychopharmacol Biol Psychiatry. (2019) 89:361–79. 10.1016/j.pnpbp.2018.10.010 30342071

[57] Jones T Moller MD. Implications of hypothalamic–pituitary–adrenal axis functioning in posttraumatic stress disorder. J Am Psychiatr Nurses Assoc. (2011) 17:393–403. 10.1177/1078390311420564 22142976

[58] Thomas N Gurvich C Kulkarni J. Borderline personality disorder, trauma, and the hypothalamus–pituitary–adrenal axis. Neuropsychiatr Dis Treat. (2019) 15:2601. 10.2147/NDT.S198804 31564884 PMC6743631

[59] Zimmerman DJ Choi-Kain LW. The hypothalamic-pituitary-adrenal axis in borderline personality disorder: a review. Harv Rev Psychiatry. (2009) 17:167–83. 10.1080/10673220902996734 19499417

[60] Wingenfeld K Spitzer C Rullkötter N Löwe B. Borderline personality disorder: hypothalamus pituitary adrenal axis and findings from neuroimaging studies. Psychoneuroendocrinology. (2010) 35:154–70. 10.1016/j.psyneuen.2009.09.014 19837517

[61] Toufexis D Rivarola MA Lara H Viau V. Stress and the reproductive axis. J Neuroendocrinol. (2014) 26:573–86. 10.1111/jne.12179 25040027 PMC4166402

[62] Vitoratos N Papatheodorou DC Kalantaridou SN Mastorakos G. “Reproductive” corticotropin-releasing hormone. Ann N Y Acad Sci. (2006) 1092:310–18. 10.1196/annals.1365.029 17308156

